# Amelioration of cognitive impairment using epigallocatechin-3-gallate in ovariectomized mice fed a high-fat diet involves remodeling with *Prevotella* and Bifidobacteriales 

**DOI:** 10.3389/fphar.2022.1079313

**Published:** 2023-01-04

**Authors:** Yang Qu, Yan Wu, Wei Cheng, Dongyang Wang, Lu Zeng, Yanru Wang, Tingting Li, Liye Zhang, Jinan Yang, Liyang Sun, Jing Ai

**Affiliations:** Department of Pharmacology, The State-Province Key Laboratories of Biomedicine-Pharmaceutics of China, College of Pharmacy of Harbin Medical University, Harbin, Heilongjiang, China

**Keywords:** estrogen deficiency, epigallocatechin-3-gallate (EGCG), gut microbiota, HFD, cognitive decline

## Abstract

**Background:** Estrogen deficiency and a high-fat diet (HFD) are both risk factors for Alzheimer’s disease (AD). HFD can accelerate cognitive impairment in estrogen-deficient patients, but there is currently no effective treatment. Epigallocatechin-3-galate (EGCG) is widely studied for its anti-inflammatory, anti-cancer, and anti-neurodegeneration effects. Nevertheless, whether EGCG can ameliorate cognitive impairment in HFD-fed estrogen-deficient mice has not been studied.

**Methods and Results:** Ovariectomized (OVX) mice fed an HFD (HFOVX) for 8 weeks experienced impaired object recognition and spatial memory, but this damage was significantly attenuated by the administration of EGCG at a dose of 45 mg/kg. Through 16S rRNA gene sequencing, we found that HFOVX changed the diversity and structure of the gut microbiota in mice, which could be restored with EGCG. Further analysis showed that HFOVX exposure not only resulted in a decrease of *Alloprevotella* in Bacteroidetes, Lactobacillaceae in Firmicutes, and *Prevotella* in Bacteroidetes but also in an increase of Bifidobacteriales in Actinobacteria. EGCG effectively reversed the decrease of *Prevotella* and inhibited the increase of Bifidobacteriales but had no effect on the decrease of *Alloprevotella* or Lactobacillaceae or on the increase of *Enterorhabdus* in HFOVX mice. Additionally, using Kyoto Encyclopedia of Genes and Genomes (KEGG) enrichment analysis, we found that EGCG significantly reversed the five functional gut microbiota genes elevated by HFOVX, including iron complex transport system substrate-binding protein, iron complex transport system permease protein, 3-oxoacyl- [acyl-carrier protein] reductase, transketolase, and 8-oxo-dGTP diphosphatase.

**Conclusions:** We concluded that EGCG improved cognitive impairment in mice with estrogen deficiency exacerbated by an HFD involved a rebuilding of the disrupted gut microbiota composition.

## Introduction

Alzheimer’s disease (AD) is an insidious neurodegenerative disease with an increasing incidence in the elderly (Joe and Ringman, 2019; Zhang Y. et al., 2020). By 2050, it is expected that the number of patients with AD will have risen to 152 million worldwide (Patterson, C, 2018). Although the pathogenesis of AD is complex, clinical trials have shown that it is more prevalent in women, who have a two- or three-fold greater risk of developing AD than men of the same age (Hamson et al., 2016). This phenomenon has been attributed to the postmenopausal phase, during which estrogen levels drop considerably. There is evidence linking estrogen deficiency to cognitive impairment by its promotion of neuroinflammation, amyloid plaque formation, and activation of the NF-κB pathway (Yun et al., 2018). However, not all postmenopausal women are susceptible to neurological disease (Tamaria et al., 2013). A high-fat diet (HFD) is considered a risk factor for multiple diseases, such as gastrointestinal cancer (Tong et al., 2021), cerebrovascular disease (Zimmerman et al., 2021), and AD (Barnes and Yaffe, 2011), as well as for postmenopausal women (Naworska and Bak-Sosnowska, 2019). A previous study demonstrated that an HFD accelerated cognitive decline in estrogen-deficient rats by aggravating mitochondrial and synaptic dysfunction (Pratchayasakul et al., 2015). However, many clinical cancer patients on antiestrogen therapy or menopausal women, especially in Western countries, are accustomed to an HFD. Finding an effective treatment strategy would significantly benefit these people.

Epigallocatechin-3-galate (EGCG), the main component of catechin in green tea, has been widely studied in recent years (Singh et al., 2011; Pervin et al., 2019). Several studies have indicated EGCG’s strong antioxidant and anti-inflammatory effects (Ohishi et al., 2016), promotion of weight loss (Mak, 2012; Yang et al., 2016), and its protection against cardiovascular (Eng et al., 2018; Zeng et al., 2021) and neurodegenerative diseases (Fernandes et al., 2021; Goncalves et al., 2021); it is also efficacious in the treatment of cancer (Gan et al., 2018; Romano and Martel, 2021). Emerging evidence shows that EGCG ameliorates the pathological changes of AD. For example, EGCG reduces levels of Aβ in the cortex and hippocampus of APP/PS1 transgenic mice, which is a traditional animal model of AD and improved behavioral deficits (Walker et al., 2015). Additionally, our previous study showed that EGCG improves cognitive impairment in 5-month estrogen-deficient mice (Zhang et al., 2021b). Nevertheless, whether it can ameliorate cognitive impairment in HFD-fed estrogen-deficient mice has not been studied.

The main limit of the clinical application of EGCG is its low bioavailability, which leads to less absorption by the brain (Kale et al., 2010). Interestingly, several studies have demonstrated that concentrations of EGCG in the intestine are significantly higher than in other organs in rats, whether administered through gavage or intravenously (Chen et al., 1997; Kim et al., 2000). This seems to indicate that gut microbiota might be a key target of EGCG. Gut microbiota, which comprise a number of microorganism species, are involved in cognitive impairment as a sensor of diet in the pathological process of AD (Li Z. et al., 2020). It has been demonstrated that *Akkermansia muciniphila* therapy protects against cognitive impairment and amyloid pathology caused by an HFD in the APP/PS1 mouse model (Ou et al., 2020). Furthermore, EGCG prevented HFD-induced increases in the Firmicutes–Bacteroidetes ratio (Remely et al., 2017) and modulated the diversity of gut microbiota in OVX rats (Zhang B. et al., 2020). However, it is unclear whether gut microbiota reconstitution is part of the process by which EGCG ameliorates cognitive impairment—the microbiota species involved in this process remain unclear. In this study, we provide evidence that EGCG improves the cognitive decline of HFD-fed OVX mice. We found that EGCG prevented the decrease of the relative abundance of *Prevotella* in Bacteroidetes and reversed the increase of the relative abundance of *Bifidobacteria* in Actinobacteria. In addition, five functional genes of gut microbiota were found to be involved in the cognitive improvement effect of EGCG on HFD-fed OVX mice. This study is the first to propose the role of EGCG in ameliorating cognitive impairment in estrogen-deficient HFD-fed mice.

## Materials and methods

### Animals

Female C57BL/6 mice (8–13 weeks old) were supplied by Changsheng Biotechnology (Shenyang, Liaoning Province, China). All experimental animals were group-housed with food and water provided *ad libitum* under a 12 h/12 h light–dark cycle and at temperatures of 23 ± 1°C with 55 ± 5% humidity. Ovariectomy surgery was performed as previously described (Zhang et al., 2021a; Zhang et al., 2021b). The mice were anesthetized with sodium pentobarbital (0.08 ml/10 g) by intraperitoneal injection. A dorsal incision was made, and the ovary, oviduct, and top of the fallopian tubes were clamped and removed. One week after surgery, an HFD and EGCG were administered for 8 weeks. The mice were then subjected to behavioral and gut microbiota analysis. In this experiment, the animals were divided into three groups: sham mice with a normal chow diet (SNCD), ovariectomized (OVX) mice fed an HFD (HFOVX), and HFOVX + EGCG mice in which HFOVX mice were treated with 5 mg/kg, 15 mg/kg, or 45 mg/kg EGCG. All experimental procedures were performed in accordance with the guidelines established by the National Institutes of Health for the care and use of laboratory animals and were approved by the Institutional Animal Care and Use Committee of Harbin Medical University (China).

### Composition of the HFD

Animal feed (45% high fat) was purchased from Xietong Pharmaceutical Bioengineering Co., Ltd. (Nanjing, Jiangsu Province, China). The HFD was composed of lard 17.5%; sucrose 12%; whole milk powder 10%; casein 13%; experimental animal premix 2%; calcium hydrogen phosphate 2%; maintenance feed 43.5%—the normal chow diet (NCD) used for the control group (SNCD mice).

### Application of EGCG

EGCG (>98% purity) was purchased from Sigma (catalog #E4143). One week after OVX surgery, mice were administered EGCG (5, 15, or 45 mg/kg body weight) dissolved in 0.9% sterile saline daily by gavage for 8 weeks.

### Behavioral tests

#### Novel object recognition

The novel object recognition experiment was performed as in previous studies (Leger et al., 2013; Zhu et al., 2018). The trial consisted of two phases over 3 days, including a habituation period on the first day, a familiarization period on the second day, and a testing period on the third day. On the first day, mice were allowed to move freely in an open field (40 cm × 40 cm × 30 cm) for 10 min to habituate to the environment. On the second day, mice were allowed to freely explore two identical objects (A and B) for 10 min, which were placed in a fixed position in an open field. On the third day of the testing period, one of the familiar objects was replaced with a novel object (C), which was completely different in color and shape from A and B, and the contact time of the mice to objects A, B, or C in the open field within 10 min was recorded. The trial in each period was performed only once for each mouse, and the field was then cleaned with 75% alcohol and was air dried naturally to eliminate any odor after each period of the trial. The Super Maze video-tracking system (Xinran, Shanghai, China) based on nose-point detection was used to record the time spent exploring objects. Active exploration was defined as mice sniffing or touching an object when the gap between the nose and object was less than 2 cm. Object preference was calculated using the following formula: preference % = [(time to explore the individual object/total exploration time for both objects) × 100%].

#### Barnes maze

The Barnes maze method has been described previously (Pitts, 2018); here, it consisted of an exposed, elevated, bright, circular white platform (91 cm in diameter) surrounded by 20 evenly spaced holes (5 cm in diameter), one of which led to an escape tunnel. The experiment was divided into four phases: adaptation, training (Days 1–5), rest (Days 6–8), and probe trials (Day 9). During the adaptation phase, the mice were placed in the escape tunnel for 1 min, and they then explored the center of the platform until they entered the escape tunnel or explored, for 5 min. After each trial, the platform and tunnel were cleaned with 75% alcohol to eliminate odor and air-dried naturally. During the training phase, the mice were placed in two randomly selected quadrants for two rounds of experiments, with an interval of 1 h. In each trial, bright light, noise, and wind were used to induce the escape behavior of mice. At the beginning of each trial, the mice were placed in a chamber located at the center of one of the four quadrants. After 15 s, the chamber was lifted to allow the mice to explore the maze. The trial stopped once the mice found the escape tunnel within 3 min. However, if a mouse failed to find the escape tunnel within 3 min, it was placed into the tunnel to adapt for an extra 20 s. In the probe trials, the escape tunnel was removed, the mice were placed in the middle of the platform for 10 s of exploration, and the trial was stopped when the heads of the mice entered the target area. For each trial, the number of incorrect holes checked (error times) and the latency time to find the escape tunnel were recorded to evaluate episodic memory.

### Gut microbiota 16S recombinant RNA (rRNA) gene sequencing

Before collecting samples, the fecal collection cage was wiped with 75% alcohol. After the mice had defecated naturally in the collection cage, the fresh feces were collected into a sterile cryopreservation tube. Samples were labeled and transferred into liquid nitrogen. The sampling was completed within 2 h and stored at −80°C prior to analysis.

### DNA extraction

The total fecal DNA of mice was extracted using a QIAamp DNA Stool Mini Kit (Qiagen, Hilden, Germany) according to the manufacturer’s protocol. Briefly, 1 ml of EX inhibition buffer and an appropriate amount of glass beads (0.5 mm diameter, Qiagen) were added per 200 mg of feces; the mixture was then homogenized at 60 Hz twice per minute with a homogenizer (FASTPREP-24, Aosheng Biotech, China). The concentration of DNA was quantified using a NanoDrop 2000 spectrophotometer (Thermo Scientific, MA, United States).

### 16S rRNA gene sequencing

16S rRNA gene amplification and sequencing were performed according to a previous study (Qian et al., 2018). Briefly, the V3–V4 regions of the 16S rRNA gene were used for PCR amplification using a protocol of 95°C for 3 min, followed by 30 cycles at 98°C for 20 s, 58°C for 15 s, and 72°C for 20 s, and a final extension at 72°C for 5 min. Universal primers were used for the quantitative real-time (qRT)-PCR primer sequences: 341F (5′-CCTACGGGRSGCAGCAG-3′) and 806R (5′-GGACTACVVGGGTATCTAATC-3′). All quantified amplicons were pooled to equalize concentrations and sequenced using an Illumina NovaSeq PE250 system (Illumina, Inc., CA, United States).

### Processing the sequencing data

The 16S rRNA gene data were processed by UPARSE to form operational taxonomic units (OTUs) with 97% similarity. A Venn diagram was used to reflect the OTU distribution in each group. The α-diversity analysis reflected the diversity of a single sample. The β-diversity was used as a criterion for intergroup microflora structure, which was determined using principal coordinates analysis (PCoA) and non-metric multidimensional scaling (NMDS) based on unweighted UniFrac distances. LDA effect size (LEfSe) analysis was used to search for species with intergroup differences among the three groups of mice: SNCD, HFOVX, and HFOVX + EGCG. Finally, the composition and abundance changes of microbiota in the three groups of samples were analyzed by different taxonomic levels.

### Statistical analysis

Data are presented as the mean and standard error of the mean (SEM). The two-tailed Student’s *t* test was applied for comparisons between two groups. Two-way ANOVA was used for comparisons of more than two groups. Tukey *post hoc* tests were used to investigate *post hoc* analyses with significant main effects. Significance was accepted at *p* < 0.05. Graphs were generated with GraphPad Prism 8.0 software (La Jolla, CA, United States).

## Results

### EGCG rescues cognitive impairment in HFOVX mice

**FIGURE 1:**
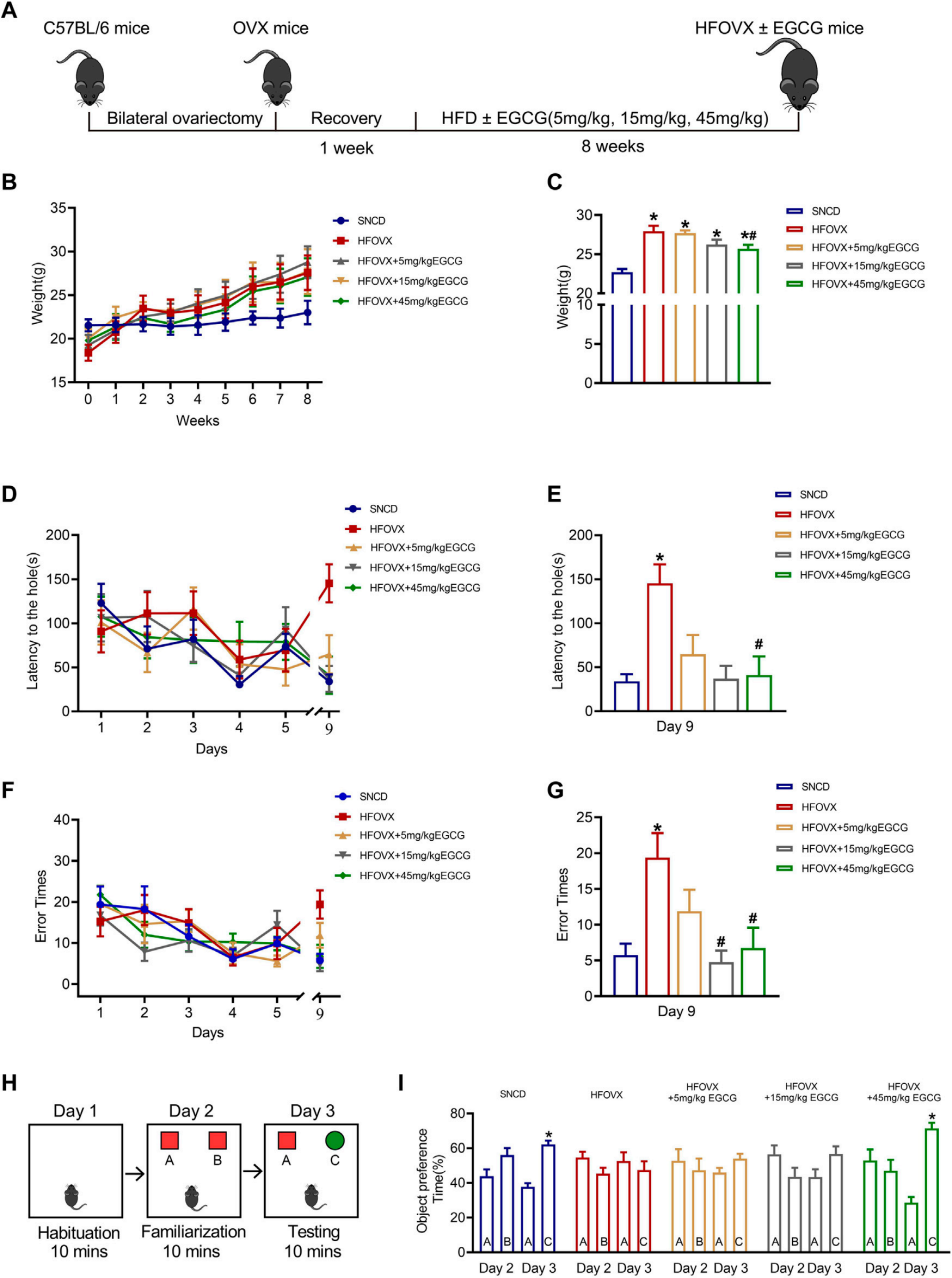
Effects of epigallocatechin-3-galate (EGCG) on body weight and cognitive function in HFOVX mice. **(A)** Schematic diagram of the experimental design, HFD, and different doses of EGCG administration. **(B)** Average weekly weight of each group. **(C)** Changes in body weight at the 8th week. *p < 0.05 HFOVX group vs. SNCD group; ^**#**^ *p* < 0.05 HFOVX+45 mg/kg EGCG group vs. HFOVX group; *n* = 7 mice. **(D)** Latency of each group of mice during the learning and testing periods. **(E)** Latency of each group of mice on the test day. **(F)** Number of errors in each group of mice during the learning and testing periods. **(G)** Error times of each group of mice on the test day. *p < 0.05 HFOVX group vs. SNCD group; ^#^ *p* < 0.05 HFOVX + EGCG group vs. HFOVX group; *n* = 8 mice. **(H)** Graphical representation of novel object recognition. **(I)** Percentage of time spent exploring the same objects on Day 2 and different objects on Day 3. *p < 0.05 preference time for C vs. A, *n* = 8 mice.

To construct the estrogen-deficient mouse model, we performed ovariectomies on female mice, as reported in our previous studies (Zhang et al., 2021a; Zhang et al., 2021b). Following a week of recovery, OVX mice were fed an HFD together with EGCG gavage therapy for 8 weeks (Figure 1A). As illustrated in Figure 1B, the body weight of HFOVX mice was much higher than that of the SNCD mice over time. At the end of the 8-week period, we found that the body weight of HFOVX mice treated with 45 mg/kg of EGCG was significantly lower than that of HFOVX mice (Figure 1C), while treatments with EGCG at doses of 5 mg/kg and 15 mg/kg did not affect the weight gain of HFOVX mice (Figure 1C). These data suggest that 45 mg/kg EGCG has a significant effect on body-weight gain in OVX mice fed an HFD.

To evaluate the effect of EGCG on the cognition of HFOVX female mice, we first performed the Barnes maze test to evaluate the action of EGCG on spatial memory. As predicted, HFOVX mice showed longer latency time (Figures 1D, E) and had more error times (Figures 1F, G) in finding the escape hole in the probe trial than did SNCD mice. In comparison with the HFOVX mice, we found that EGCG administration at 5 mg/kg shortened the latency time for finding the escape hole (Figure 1D), although it had no effect on the increased error times of HFOVX mice (Figure 1G). Furthermore, when HFOVX mice were treated with EGCG at doses of 15 mg/kg or 45 mg/kg, we found that EGCG significantly reversed both the prolonged latency time (Figure 1E) and increased error times (Figure 1F) for finding the escape hole compared to HFOVX mice. Considering that the Barnes maze test could not determine the impairment of episodic memory, we performed a novel object recognition experiment to evaluate the episodic memory (Figure 1H). Unlike the SNCD mice, HFOVX mice showed no preference for new object C on Day 3 (Figure 1I). Unlike the HFOVX mice, HFOVX mice treated with EGCG at a dose of 45 mg/kg showed an obvious preference for new object C on Day 3, which was similar to the behavior of SNCD mice (Figure 1I), but EGCG administered at doses of 5 mg/kg and 15 mg/kg did not affect the aversion to novel object C in HFOVX mice (Figure 1I). The results together indicate that 45 mg/kg of EGCG had a more stabilizing effect on ameliorating cognitive decline than did the 5 mg/kg and 15 mg/kg doses of EGCG. Therefore, we chose 45 mg/kg of EGCG to explore the mechanism for improving cognitive function in HFOVX mice.

### EGCG improves β-diversity rather than α-diversity of gut microbiota in HFOVX mice

**FIGURE 2:**
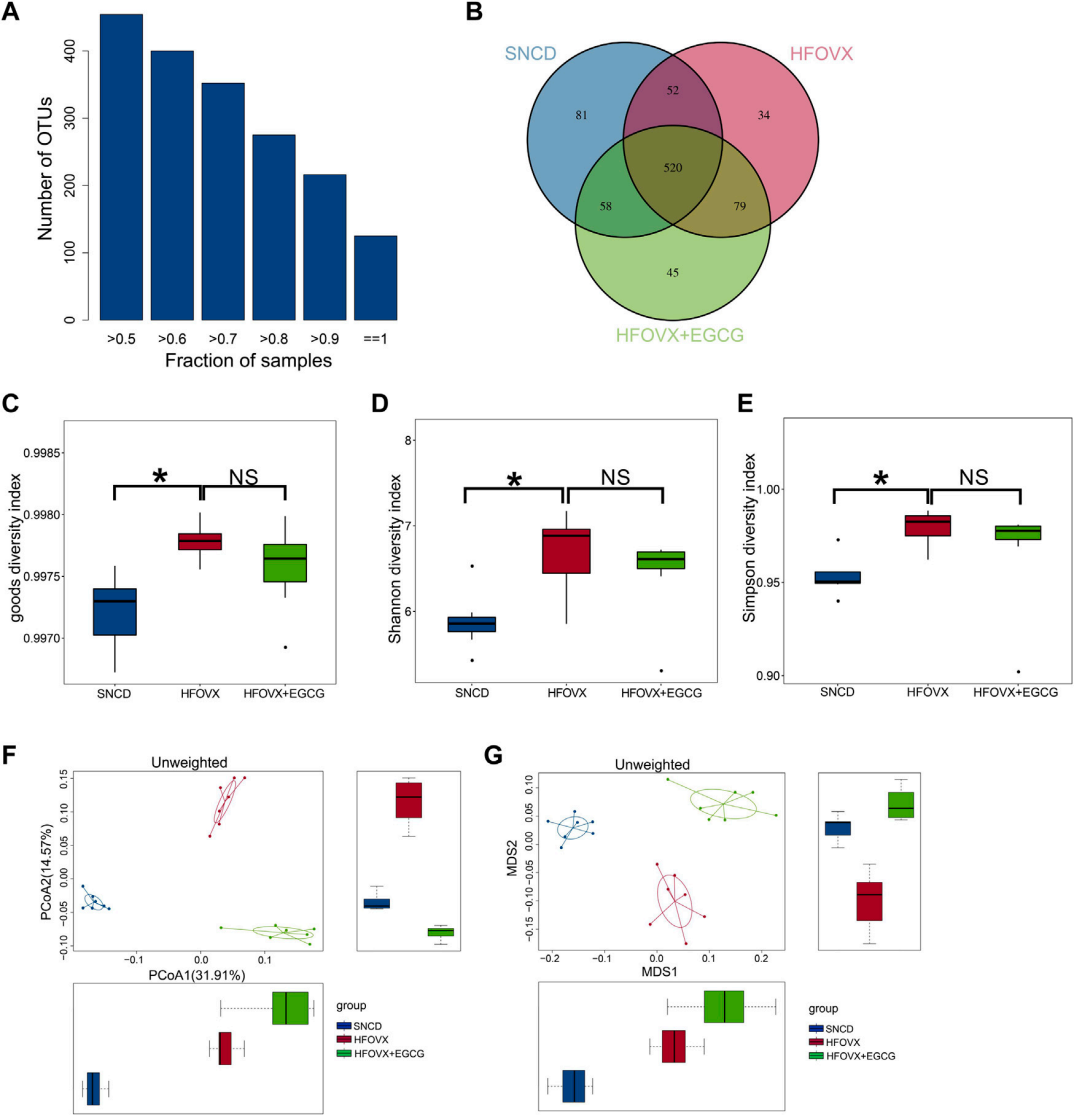
Principal component analysis of OTUs for gut microbiota with different treatments. **(A)** Relationship between the total OTU number and sample number. The x-coordinate is the proportion of the covered samples, and the y-coordinate is the number of OTUs for which coverage is greater than the sample proportion. **(B)** OTUs on a Venn diagram with different color graphics representing different samples (groups); the overlapping region of different colors is the number of OTUs common across samples (groups). Blue represents the OTU number of the SNCD mice; red represents the OTU number of the HFOVX mice; green represents the OTU number of the HFOVX+45 mg/kg EGCG mice; *n* = 7 mice. **(C–E)** Goods coverage **(C)**, Shannon **(D)**, and Simpson **(E)** diversity indexes. The abscissa represents the sample groups, and the ordinate represents the goods coverage, Shannon, and Simpson index values for the different groups. *p < 0.05, HFOVX group vs. SNCD group; *n* = 7 mice. NS: no statistical significance. **(F–G)** Unweighted principal coordinates analysis (PCoA) **(F)** and non-metric multidimensional scaling (NMDS) **(G)** based on the distance matrix of UniFrac dissimilarity of the gut microbial communities in mice. Each point represents one sample, and each group is denoted by a different color. Circles combine samples from the same group by their respective 95% confidence interval ellipses; *n* = 7 mice.

Previous studies have demonstrated that gut microbiota homeostasis is crucial for memory and cognitive function (Gareau, 2014; Wang et al., 2019). We performed 16S rRNA gene sequencing to evaluate whether EGCG-improved cognitive function was related to its influence on intestinal microbiota homeostasis by comparing the gut microbiota composition in the SNCD, HFOVX, and HFOVX + EGCG mice (45 mg/kg). First, we obtained the number of OTUs for each sample based on 97% similarity (Figure 2A). We found that 520 OTUs were shared by all three groups of mice; however, 52, 58, and 79 OTUs were unique to the SNCD, HFOVX, and HFOVX + EGCG mice, respectively (Figure 2B). These results suggest that EGCG treatment changed the general gut microbiota composition of HFOVX mice. We next observed the changes in gut microbiota diversity and internal structure by analyzing the α- and β-diversities. The mean community diversity indexes (i.e., the α-diversity, including goods coverage and Shannon and Simpson indexes, based on OTU levels) of the HFOVX mice were significantly higher than those of the SNCD mice, but EGCG administration had no effect on the α-diversity of HFOVX mice (Figures 2C–E). It is noteworthy that, based on the unweighted UniFrac analysis (Figure 2F) and NMDS analysis (Figure 2G), we found that the β-diversity was significantly lower in HFOVX mice than SNCD mice and that this effect was reversed by EGCG treatment (Figures 2F, G). Taken together, these results indicate that EGCG rescued the gut microbiota disruption in HFOVX mice.

### EGCG administration affects the abundance of Bacteroidetes*,* Actinobacteria*,* Firmicutes, and Verrucomicrobia in HFOVX mice

**FIGURE 3:**
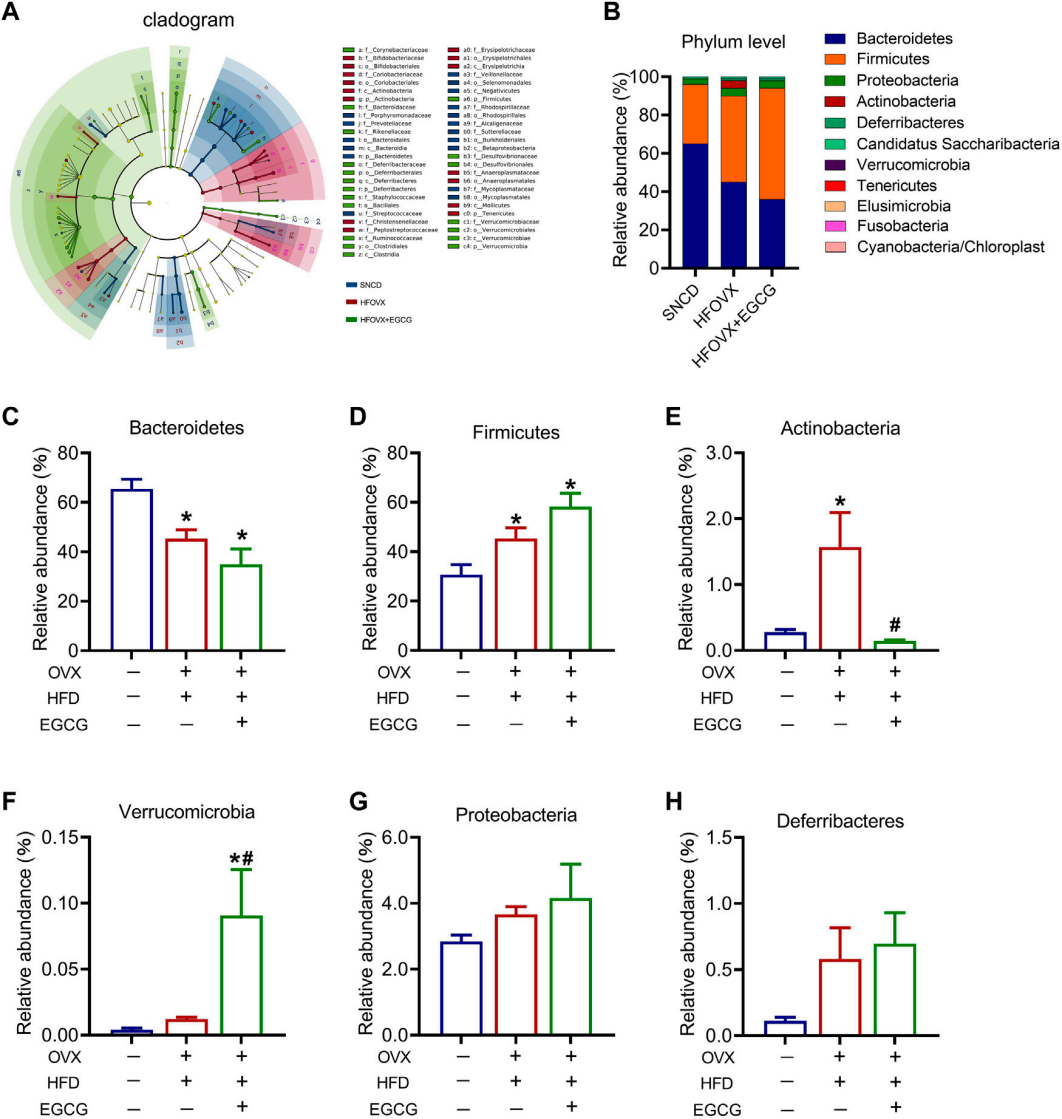
Influence of an HFD with estrogen deficiency and EGCG treatment on gut microbiota. **(A)** Cladogram of linear discriminant analysis (LDA) effect size (LEfSe) method shows the phylogenetic distribution of gut microbiota in the three groups of mice. Blue represents the SNCD group; red represents the HFOVX group; green represents the HFOVX+45 mg/kg EGCG group. **(B)** Species profiling histogram of the gut microbiota community structure at the phylum level in each group. The abscissa is the group name, and the ordinate is the relative abundance of species. Different colors correspond to different species names, and the length of the color block indicates the relative abundance of the species represented by the color block; *n* = 7 mice. **(C–H)** Relative abundance of each group at the phylum level of Bacteroidetes **(C)**, Firmicutes **(D)**, Actinobacteria **(E)**, Verrucomicrobia **(F)**, Proteobacteria **(G),** and Deferribacteres **(H)**. *p < 0.05 HFOVX group vs. SNCD group, or HFOVX + EGCG vs. SNCD group; ^**#**^ *p* < 0.05 HFOVX + EGCG vs. HFOVX group; NS: no statistical significance, *n* = 6–7 mice.

To better understand how EGCG affected the specific differential gut microbiota in the HFOVX mice, we utilized LEfSe analysis to identify the presence and effect of species with significant differences (Segata et al., 2011; Eren et al., 2013). We observed that, although the composition of gut microbiota differed among SNCD, HFOVX, and HFOVX + EGCG mice, the most abundant microbiota were Bacteroidetes, Actinobacteria, Firmicutes, Verrucomicrobia, Deferribacteres, and Proteobacteria (Figure 3A). We next considered the differences in the taxa at the phylum level and found that, compared with the SNCD group, HFOVX altered the intestinal microbial communities, as evidenced by the decrease in Bacteroidetes and increase in Actinobacteria (Figures 3B–E)—although it did not affect the abundance of Verrucomicrobia, Deferribacteres, or Proteobacteria (Figures 3F–H). Interestingly, we found that EGCG treatment did not affect the abundance of either Bacteroidetes or Firmicutes (Figures 3C,D); however, EGCG administration strongly reversed the increased abundance of Actinobacteria (Figure 3E). Considering the content and physiological effect of Verrucomicrobia, Deferriobacteria, and Proteobacteria, we also evaluated the changes in their relative abundance among the three groups of mice. EGCG treatment significantly increased the relative abundance of Verrucomicrobia in HFOVX mice (Figure 3F). However, there were no significant differences in the relative abundance of Proteobacteria and Deferriobacteria among the three groups (Figures 3G, H). Together, these results suggested that the changes in the relative abundance of Bacteroidetes, Actinobacteria, Firmicutes, and Verrucomicrobia may be involved in the ameliorating effect of EGCG on the cognitive decline of HFOVX mice.

### *Prevotella* and Bifidobacteriales are key targets for EGCG-mediated amelioration of cognitive impairment in HFOVX mice

**FIGURE 4:**
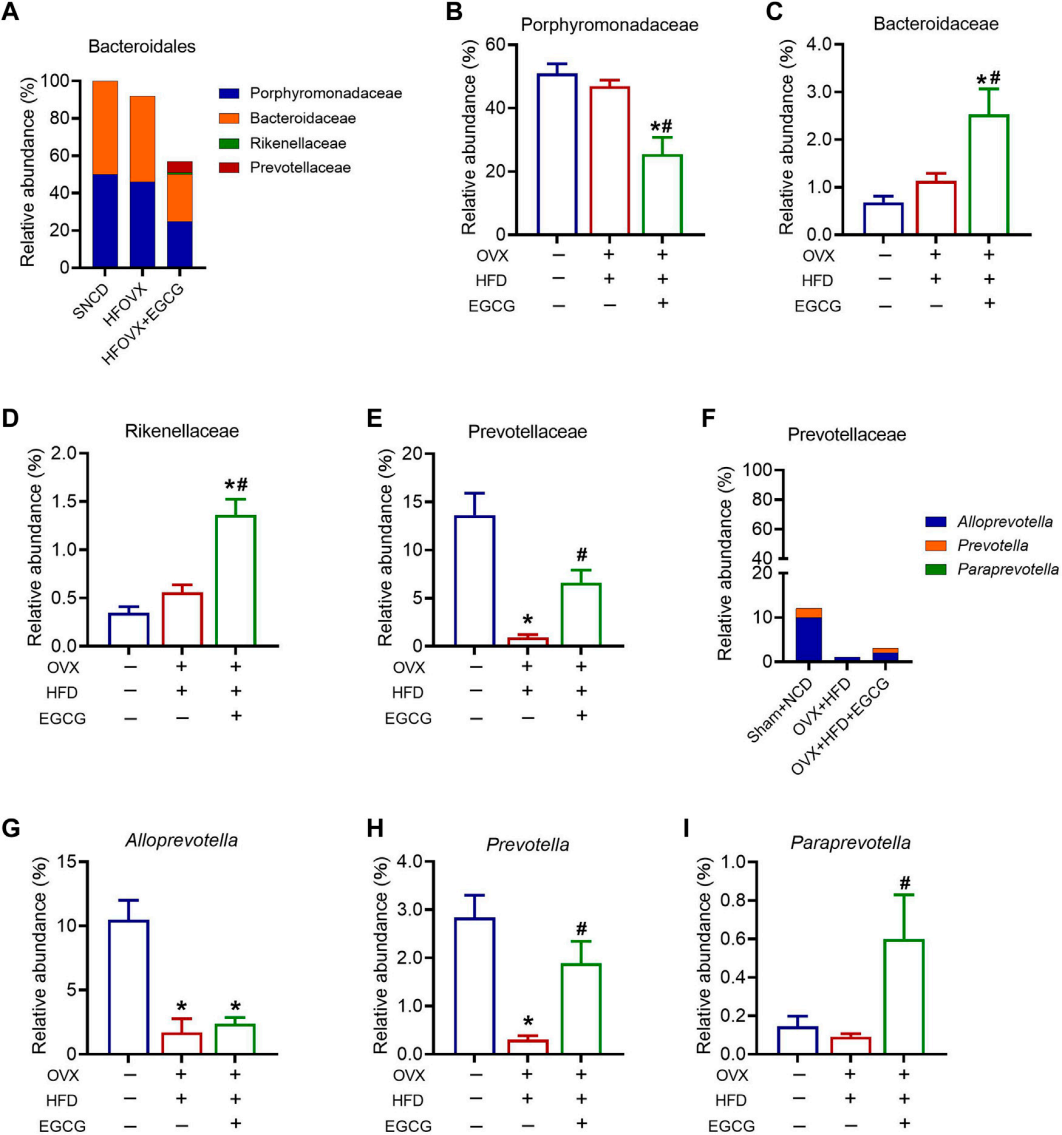
Different abundances of gut microbiota at the Bacteroidetes level. **(A)** Species profiling histogram of the gut microbiota community structure of Bacteroidales in each group. **(B–E)** Relative abundance of Porphyromonadaceae **(B)**, Bacteroidaceae **(C)**, Rikenellaceae **(D)**, and Prevotellaceae **(E)** across the three groups. **(F)** Species profiling histogram of gut microbiota community structure of Prevotellaceae in each group. **(G–I)** Relative abundance of *Alloprevotella* **(G)**, *Prevotella* **(H)**, and *Paraprevotella* **(I)** among the three groups. **p < 0.01, ***p < 0.001 HFOVX group vs. SNCD group, or HFOVX + EGCG vs. SNCD group; ^**#**^ *p* < 0.05, HFOVX + EGCG vs. HFOVX group, NS: no statistical significance; *n* = 6–7 mice.

We next sought to identify specific gut bacteria that belong to the phyla of Bacteroidetes, Actinobacteria, Firmicutes, and Verrucomicrobia, at the class, order, family, and genus levels among the three groups. We first analyzed the relative abundance of Porphyromonadaceae, Bacteroidaceae, Rikenellaceae, and Prevotellaceae, which belong to Bacteroidetes (Figure 4A). The results showed that the relative abundances of Porphyromonadaceae, Bacteroidellaceae, and Rikenellaceae did not change significantly in the HFOVX mice compared with the SNCD mice (Figures 4B–D). However, EGCG administration significantly altered the reduced relative abundance of Porphyromonadaceae (Figure 4B) and elevated the relative abundances of Bacteroidellaceae (Figure 4C) and Rikenellaceae (Figure 4D). Importantly, compared with SNCD mice, the relative abundance of Prevotellaceae decreased remarkably in HFOVX mice, which was reversed by EGCG treatment (Figure 4E). Second, we analyzed the relative abundance of Prevotellaceae in the taxa at the family level, which includes *Alloprevotella*, *Prevotella*, and *Paraprevotella* (Figure 4F). We found that, compared with the SNCD mice, the relative abundance of *Alloprevotella* and *Prevotella* decreased significantly in HFOVX mice (Figures 4G, H). Further study showed that EGCG treatment reversed the decreased relative abundance of *Prevotella* and increased the abundance of *Paraprevotella* in HFOVX mice (Figures 4H, I), although there was no effect on the relative abundance of *Alloprevotella* (Figure 4G). These results implied that the reversal effect of EGCG on the cognitive impairment of the HFOVX mice might be related to the increase in the relative abundance of Prevotellaceae in Bacteroidetes, which was mainly dependent on *Prevotella* but not related to the relative abundance of Porphyromonadaceae*,* Bacteroidaceae, or Rikenellaceae in Bacteroidetes.

**FIGURE 5:**
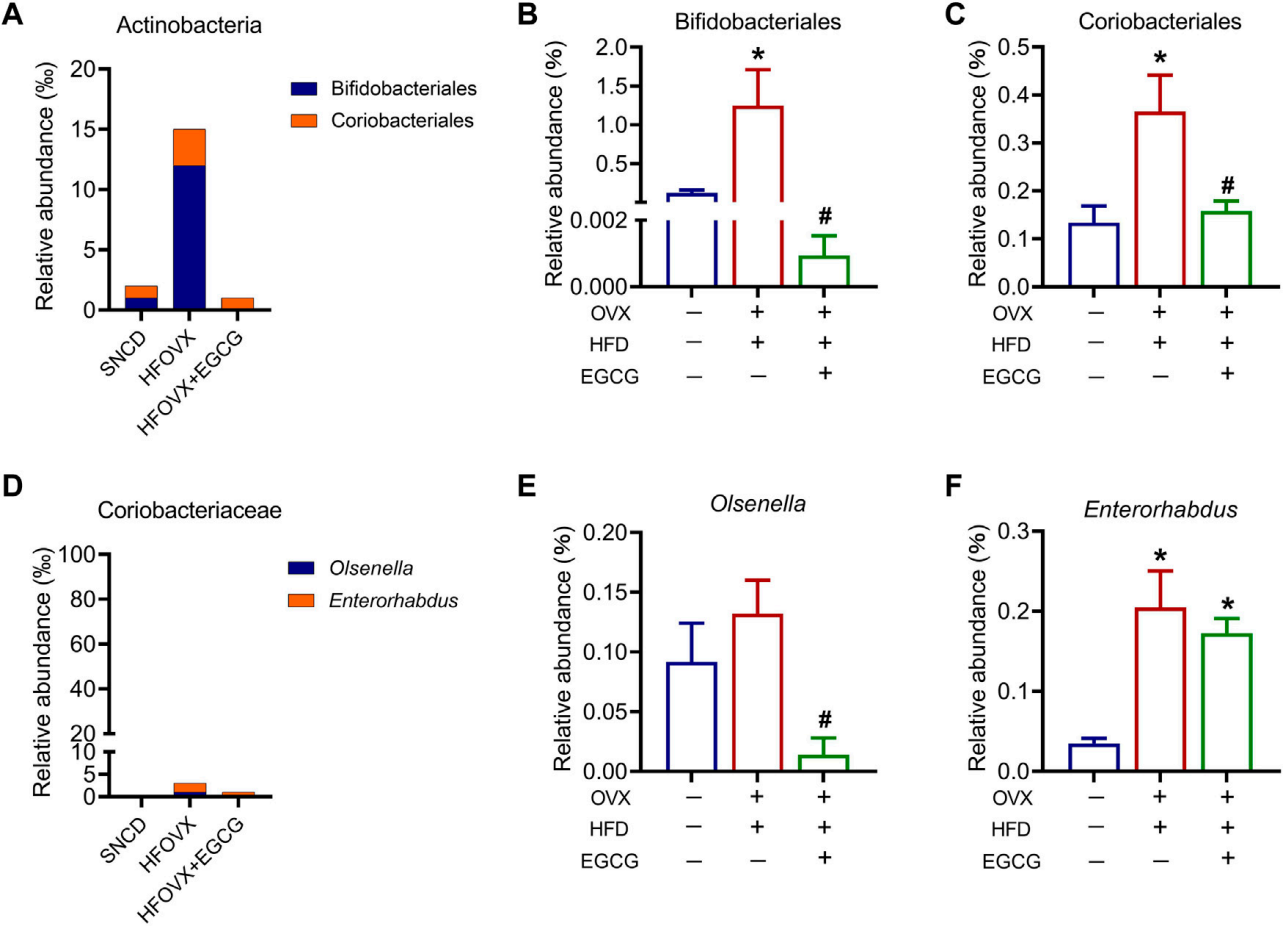
Effects of EGCG on the relative abundance of Actinobacteria in HFOVX mice. **(A)** Species profiling histogram of the relative abundance of the Actinobacteria community structure in each group, including Bifidobacteriales **(B)** and Coriobacteriales **(C)**. **(D–F)** Relative abundance of the Coriobacteriales **(D)**, *Olsenella* **(E)**, and *Enterorhabdus* levels **(F)** in SNCD, HFOVX and HFOVX + EGCG mice. *p < 0.05, HFOVX group vs. SNCD group, or HFOVX + EGCG vs. SNCD group; ^**#**^ *p* < 0.05 HFOVX + EGCG vs. HFOVX group; *n* = 6–7 mice.

We next analyzed the relative abundance of Actinobacteria in the taxa at the order level. Because the relative abundance of Actinomycetales was very low, we only investigated the changes in the relative abundance of Bifidobacteriales and Coriobacteriales (Figure 5A). We found that the relative abundance of Bifidobacteriales and Coriobacteriales was significantly increased in the HFOVX mice compared with the SNCD mice and that EGCG administration reversed this phenomenon (Figures 5B, C). Since there was only one family or genus in Bifidobacteriales, the variation of relative abundance was consistent across order, family, and genus. Therefore, we only explored the relative abundance of *Olsenella* and *Enterorhabdus*, which belong to Coriobacteriales (Figure 5D). We found that there was no change in the relative abundance of *Olsenella* in HFOVX mice (Figure 5E), but the relative abundance of *Enterorhabdus* increased significantly (Figure 5F). However, EGCG administration remarkably decreased the relative abundance of *Olsenella* (Figure 5E) but did not affect the relative abundance of *Enterorhabdus* (Figure 5F) in HFOVX mice. Collectively, these results implied that Bifidobacteriales under Actinobacteria might be involved in the improvement of cognitive impairment in the EGCG + HFOVX mice.

**FIGURE 6:**
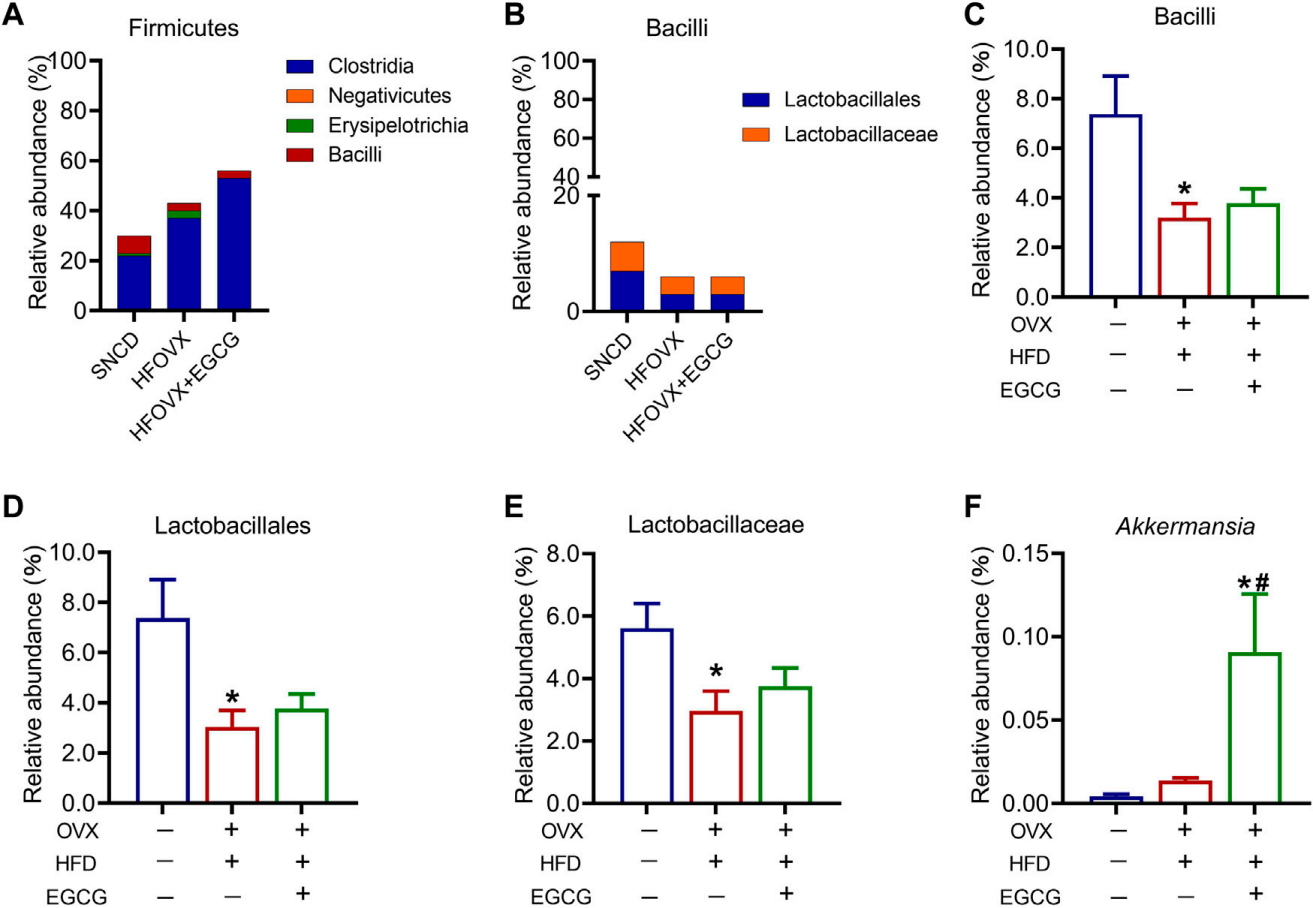
Different abundances of gut microbiota in the Firmicutes and *Verrucomicrobia*. **(A–B)** Relative abundance of the composition of Firmicutes **(A)** and Bacilli **(B)**. **(C–F)** Relative abundance of Bacilli **(C)**, Lactobacillales **(D)**, Lactobacillaceae **(E)**, and *Akkermansia* **(F)** in the three groups. *p < 0.05 HFOVX group vs. SNCD group, or HFOVX + EGCG vs. SNCD group; ^**#**^ *p* < 0.05 HFOVX + EGCG vs. HFOVX group; *n* = 6–7 mice.

We then analyzed four classes under Firmicutes—Clostridia, Negativicutes, Erysipelotrichia, and Bacilli—among the three groups (Figure 6A). Of these, *Lactobacillus* under Bacilli has been widely studied as a probiotic. Since there was only one order or family above *Lactobacillus*, the variation in relative abundance was consistent across order, family, and genus. Therefore, we focused on the analysis of the variation in relative abundance of Bacilli, Lactobacillales, and Lactobacillaceae in the mice (Figure 6B). We found that this abundance significantly decreased in the HFOVX mice compared with the SNCD mice (Figures 6C–E); however, EGCG administration failed to improve the reduction of these three gut microbiota in the HFOVX mice (Figures 6C–E). Finally, we compared the changes in the relative abundance of *Akkermansia* under Verrucomicrobia in the three groups of mice; the relative abundance in HFOVX mice did not significantly differ from the SNCD mice but was significantly increased by EGCG (Figure 6F). These data demonstrate that EGCG improves cognitive impairment in HFOVX mice independently of Bacilli, Lactobacillales, Lactobacillaceae, and *Akkermansia*.

### Five functional genes of gut microbiota are involved in the effects of EGCG

**FIGURE 7:**
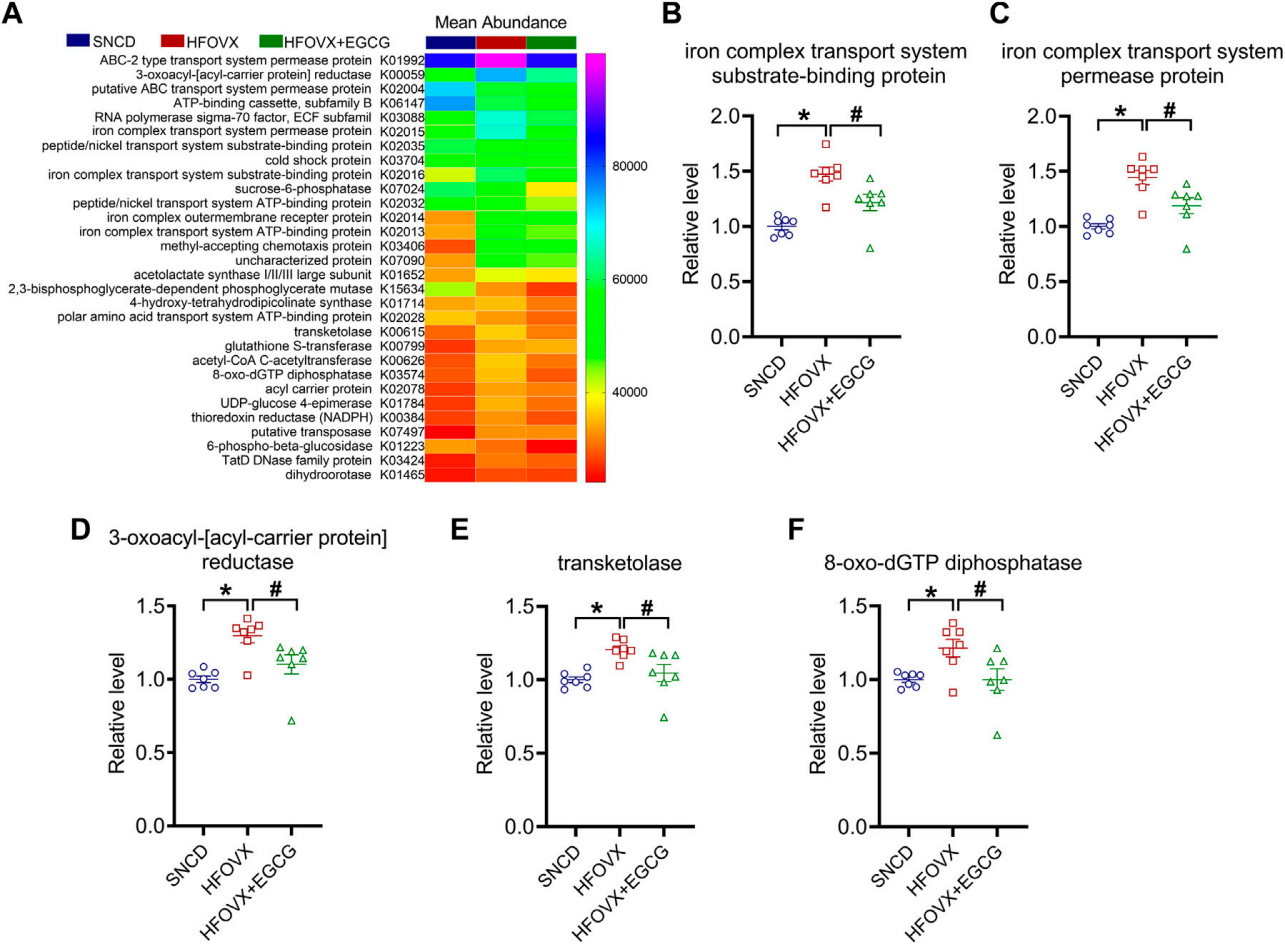
Functional gene predictions of the gut microbiota related to EGCG. **(A)** Heatmap shows the top 30 KOs closely associated with HFOVX and EGCG. **(B–F)** Relative abundance of the five functional genes of gut microbiota was significantly different. *p < 0.05, HFOVX group vs. SNCD group; ^**#**^ *p* < 0.05 HFOVX + EGCG vs. HFOVX group; *n* = 7 mice.

Finally, to confirm the functional genes of the gut microbiota associated with cognitive impairment and EGCG administration, we used Phylogenetic Investigation of Communities by Reconstruction of Unobserved States (PICRUSt) software to predict the abundance of the functional categories in the Kyoto Encyclopedia of Genes and Genomes (KEGG) Ortholog (KO) database according to a previous study (Qian et al., 2018). We selected the top 30 KOs for the following experiments (Figure 7A). As predicted, we found that gut microbiota in the HFOVX mice were enriched in genes encoding iron complex transport system substrate-binding protein, iron complex transport system permease protein, 3-oxoacyl-[acyl-carrier protein] reductase, transketolase, and 8-oxo-dGTP diphosphatase, and that all these changes were prevented by EGCG administration (Figures 7B–F). Taken together, these results implied that the improvement of cognitive impairment by EGCG might be related to these five functional genes.

## Discussion

As a strong antioxidant and anti-inflammatory molecule, EGCG was found to have satisfactory neuroprotective effects and that it therefore benefitted patients with Parkinson’s disease, AD, and ischemic brain damage (Nagai et al., 2002; Pervin et al., 2018; Velmurugan et al., 2018). This is the first study to report that EGCG can also prevent cognitive decline in OVX mice fed an HFD. By performing 16S rRNA gene sequencing, we identified that *Prevotella* in Bacteroidetes and Bifidobacteriales in Actinobacteria were the key targets of EGCG. We then found that the EGCG–gut microbiota–iron axis may mediate the therapeutic effect of EGCG on HFOVX-induced cognitive decline in mice. This study revealed a novel mechanism by which an HFD promoted cognitive decline in OVX mice and provided new potential targets for the therapeutic effect of EGCG. The results could benefit women suffering from menopause, ovarian cancer, and breast cancer on anti-estrogen therapy, who tend to consume an HFD.

Estrogen deficiency is considered to be the most important risk factor for dementia in women (Paganini-Hill and Henderson, 1994; Gandy and Duff, 2000). However, our previous study found that estrogen deficiency induced by ovariectomy successfully induced impairment of spatial memory in the fifth month after surgery (Zhang et al., 2021a). In the present study, we found that OVX mice showed a marked cognitive decline after 8 weeks when they were fed an HFD—here, the cognitive decline of OVX mice occurred at least 2 months earlier. This phenomenon indicated that an HFD exacerbate cognitive impairment due to estrogen deficiency in mice. Previous studies reported that an HFD impaired hippocampus-dependent cognition by reducing *Prevotella* in Bacteroidetes (Heisel et al., 2017; Yang et al., 2019) and *Lactobacillus* in Firmicutes (Ji et al., 2021). Moreover, the level of *Prevotella* in Bacteroidetes and *Enterorhabdus* in Actinobacteria was reduced in OVX rats (Zhang et al., 2017). These results suggested that gut microbiota homeostasis is involved in cognitive impairment due to an HFD or an estrogen deficiency. Here, we found that, when estrogen-deficient mice were fed an HFD (HFOVX), the composition of gut microbiota was different from that in mice with estrogen deficiency or in sham mice. We found that HFOVX not only led to a decrease of *Prevotella* in Bacteroidetes, *Lactobacillus* in Firmicutes, *Alloprevotella* in Bacteroidetes, and Lactobacillaceae in Firmicutes but that it also led to an increase of Bifidobacteriales in Actinobacteria. Additionally, HFOVX exposure induced a significant increase of *Enterorhabdus* in Actinobacteria, which was reported to decrease in OVX rats (Zhang et al., 2017). These results indicated that the heterogeneity of gut microbiota composition may be involved in HFD-induced cognitive impairment in OVX mice.

EGCG has been extensively studied in relation to AD due to its antioxidant and anti-inflammatory effects, as an oxidative and inflammatory state is an important neuropathological hallmark of AD (Chen and Zhong, 2014; Ganguly et al., 2021); however, its low bioavailability hinders its clinical application. The search for novel and more effective drug targets of EGCG to improve its pharmaceutical effect at lower doses is highly anticipated. Previous studies reported that the content of EGCG in the intestine was significantly higher than in other organs when EGCG was administered by gavage (Chen et al., 1997; Kim et al., 2000). This suggests that gut microbiota may be a key therapeutic target for EGCG. *Prevotella* is a common human gut microbe that has been demonstrated to be both positively and negatively correlated with the health of the host (Tett et al., 2019). The abundance of *Prevotella* negatively correlated with cognitive impairment (Wang et al., 2016; Yan et al., 2021). Additionally, *Bifidobacteria* has been reported as being positively correlated with an HDF, and as aggravating immune deficiency damage (Wu et al., 2011; Esaiassen et al., 2017). All these results suggest that *Prevotella* and Bifidobacteriales may be the targets of EGCG in the treatment of cognitive deficits induced by HFOVX exposure.

In addition, we noted no difference in the levels of *Paraprevotella* in Bacteroidetes, *Olsenella* in Actinobacteria, and *Akkermansia* in Verrucomicrobia between the SNCD and HFOVX mice, but that EGCG induced a significant decrease in *Olsenella* levels and an increase in *Paraprevotella* and *Akkermansia*. Previously, *Paraprevotella* and *Olsenella* were found to have a regulatory effect on inflammation and were associated with the occurrence of intestinal inflammation (Human Microbiome Project, 2012; Perez-Munoz et al., 2014) and were significantly reduced in stroke and transient ischemic patients (Li F. H. et al., 2020), respectively. *Akkermansia* was reported to be a mucus-degrading bacteria in the mucus layer that promoted neuronal development and plasticity in the hippocampus that protected cognitive function (Yang et al., 2019)—its mechanism requires further study. Whether *Olsenella*, *Paraprevotella*, and *Akkermansia* are also the targets of EGCG requires future evaluation.

Finally, we discovered, through KEGG enrichment analysis, five functional gut microbiota genes linked to EGCG function: iron complex transport system substrate-binding protein, iron complex transport system permease protein, 3-oxoacyl-[acyl-carrier protein] reductase, transketolase, and 8-oxo-dGTP diphosphatase. The genes related to iron complex transport system substrate-binding protein have been more studied in the development of AD than the other genes. It has been reported that iron overload promoted β-amyloid (Aβ) plaque aggregation and tau hyperphosphorylation, and ultimately impaired cognitive function (Peng et al., 2021; Wang et al., 2022)—consistent with the results observed in the HFOVX mice. Notably, we were surprised to find that iron status influenced non-alcoholic fatty liver disease in obese patients through *Prevotella* and *Bifidobacteria* (Mayneris-Perxachs et al., 2021)*.* Furthermore, strains of the *Bifidobacteria* family bind iron directly to their surface (Kot and Bezkorovainy, 1999). A recent study found that EGCG promoted neuronal regeneration and memory and cognitive function by inhibiting iron accumulation (Chen et al., 2022), thus providing evidence for our findings in the current study that EGCG significantly prevented the increase of iron-related transporters in the HFOVX mice. However, whether *Prevotella* or *Bifidobacteria* is involved in the EGCG–iron axis to ameliorate cognitive impairment remains for further investigation.

## Conclusion

Taken together, our study provides a new insight into treatment with EGCG on the cognitive deficits in mice co-exposed to estrogen deficiency and an HFD. The mechanism is associated with an EGCG-mediated increase in the relative abundance of *Prevotella* in Bacteroidetes and decrease in the abundance of Bifidobacteriales in Actinobacteria*.* We also propose that the EGCG–gut microbiota–iron axis is an important factor in attenuating cognitive impairment in HFOVX mice. However, the contribution of this axis and specific gut microbiota OTUs to the EGCG effects on HFOVX-induced cognitive impairment requires further validation.

## Data Availability

The datasets presented in this study can be found in NCBI BioProject repositories (https://www.ncbi.nlm.nih.gov/bioproject), accession number: PRJNA907178.
